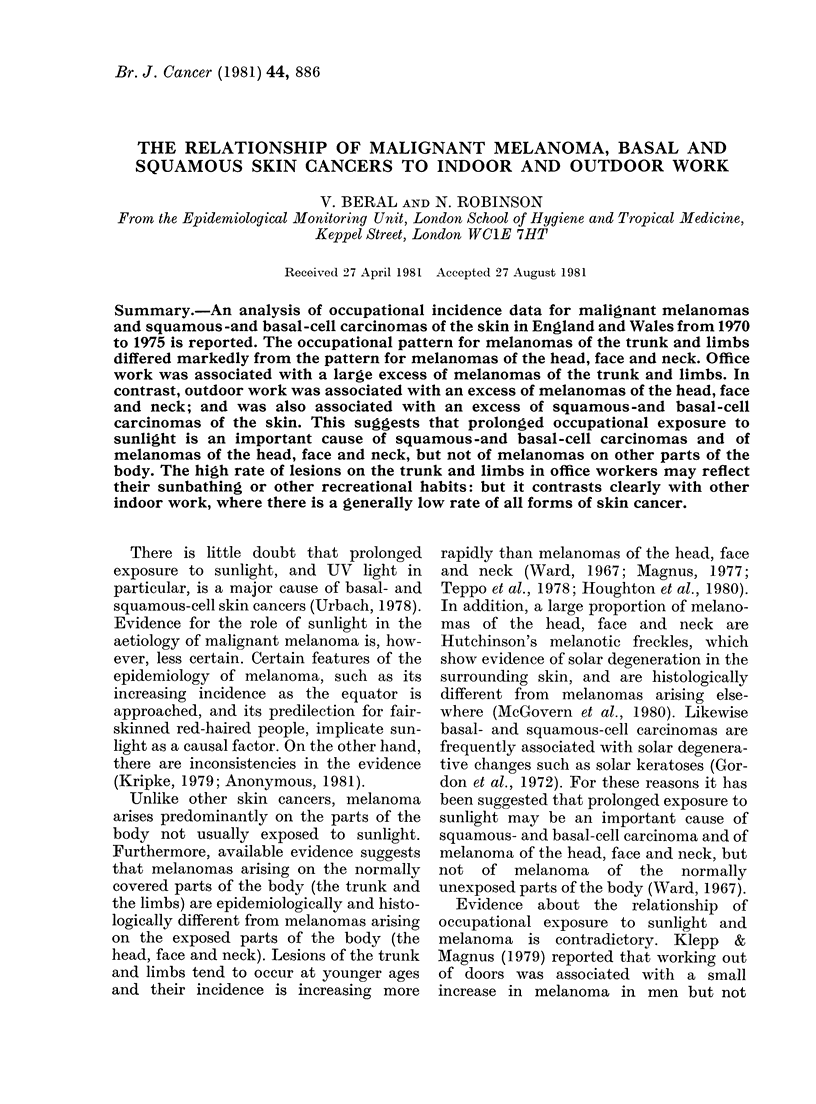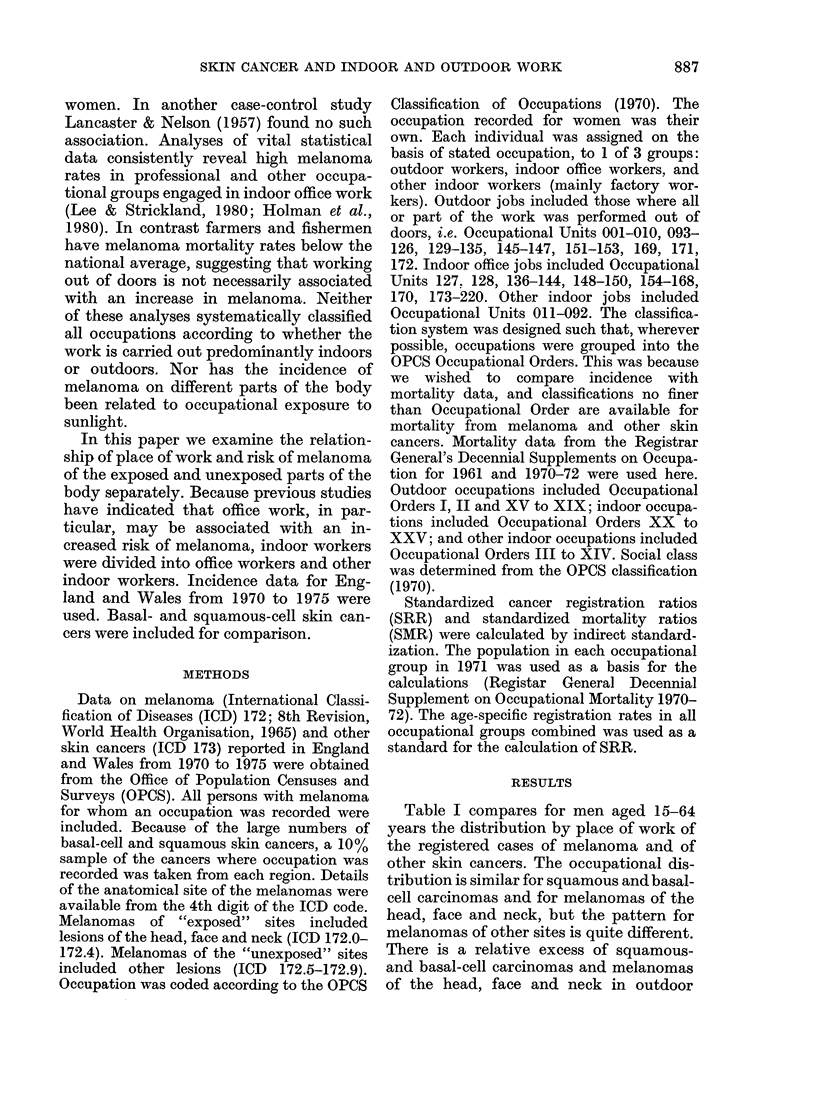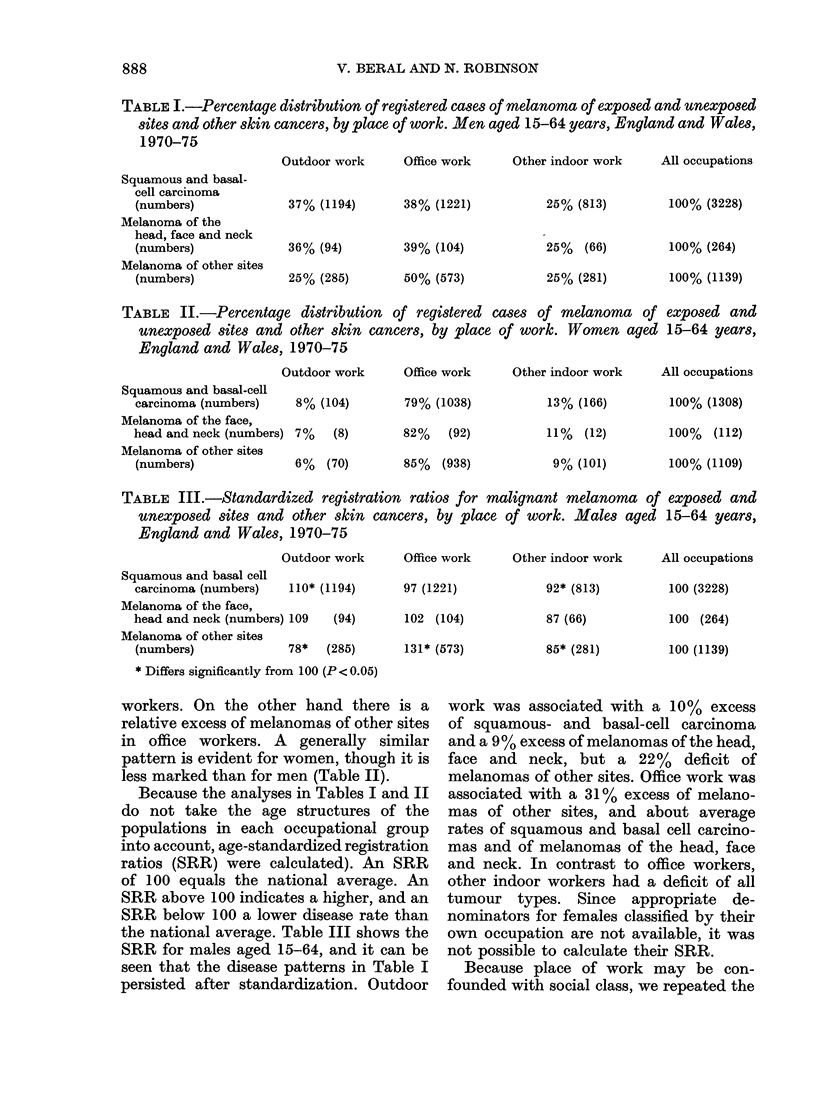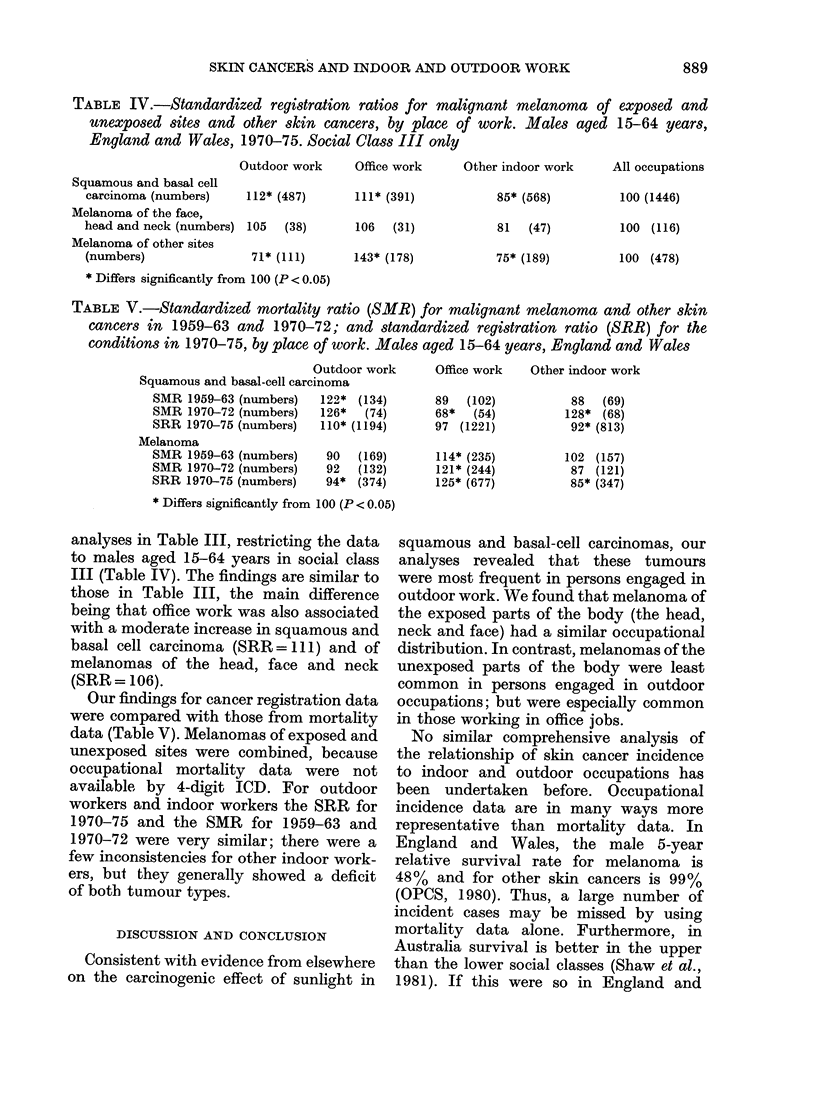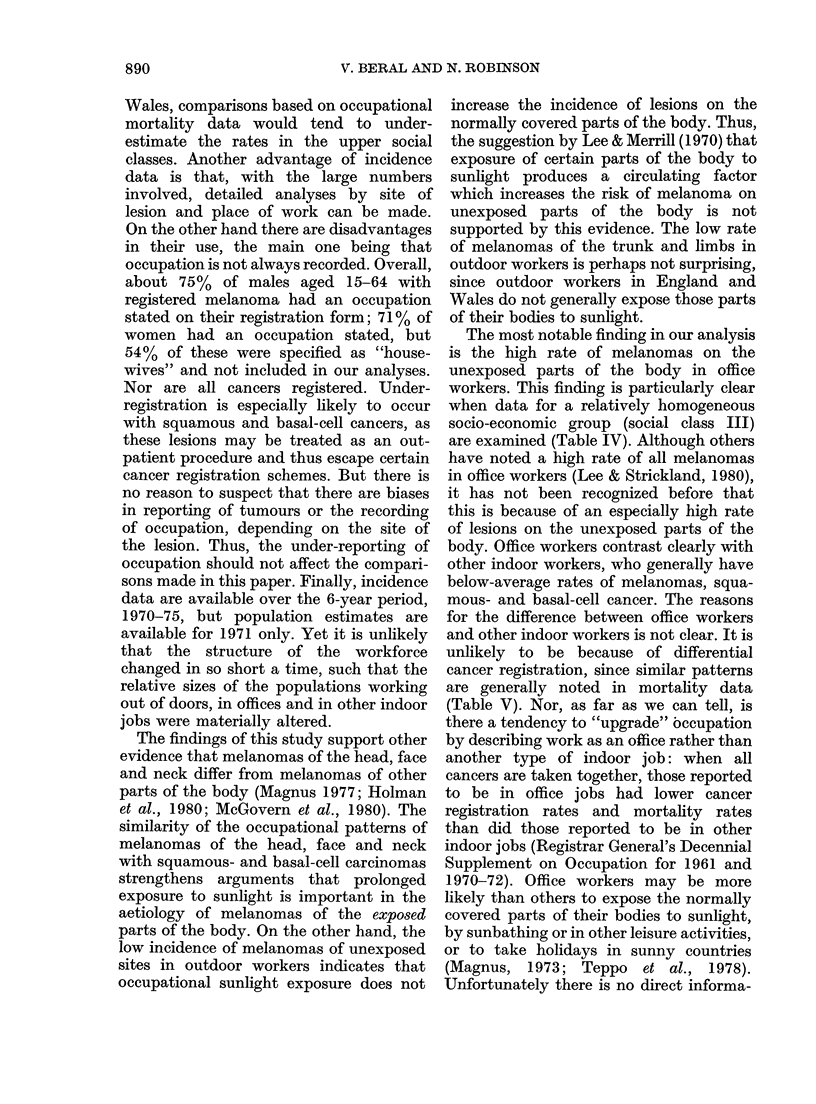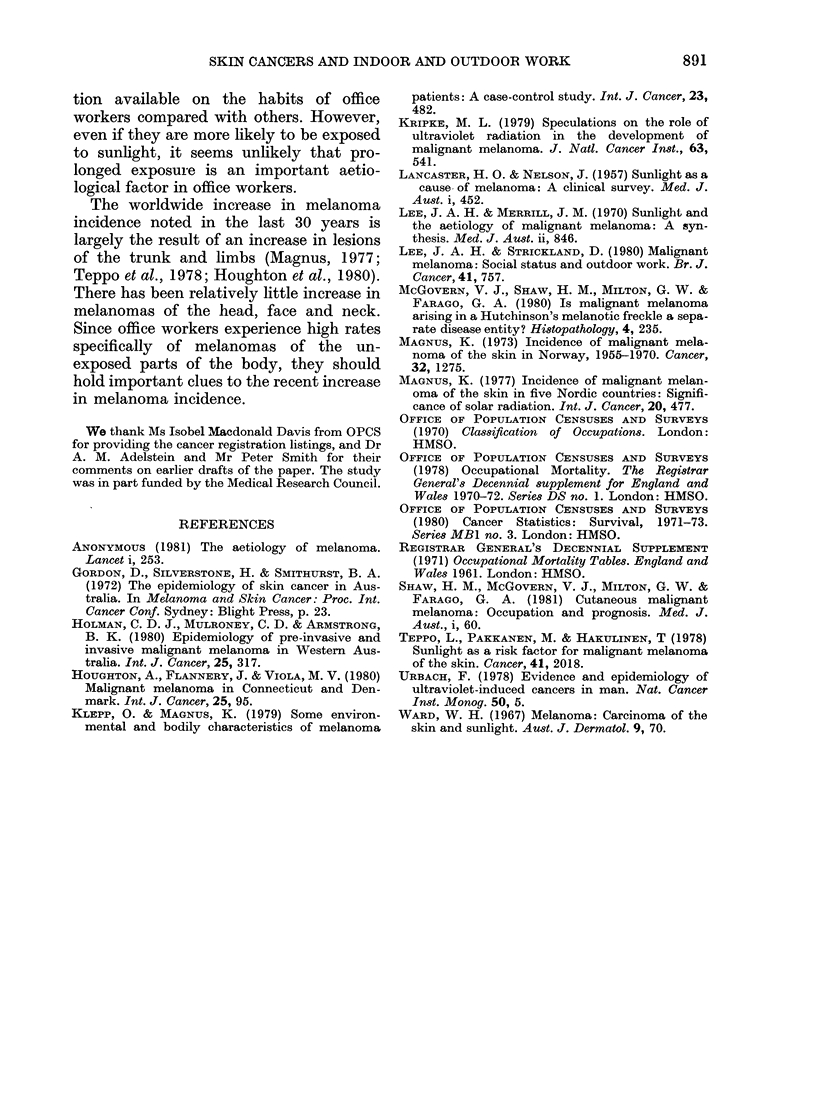# The relationship of malignant melanoma, basal and squamous skin cancers to indoor and outdoor work.

**DOI:** 10.1038/bjc.1981.288

**Published:** 1981-12

**Authors:** V. Beral, N. Robinson

## Abstract

An analysis of occupational incidence data for malignant melanomas and squamous-and basal-self carcinomas of the skin in England and Wales from 1970 to 1975 is reported. The occupational pattern for melanomas of the trunk and limbs differed markedly from the pattern for melanomas of the head, face and neck. Office work was associated with a large excess of melanomas of the trunk and limbs. In contrast, outdoor work was associated with an excess of melanomas of the head, face and neck; and was also associated with an excess of squamous-and basal-cell carcinomas of the skin. This suggests that prolonged occupational exposure to sunlight is an important cause of squamous-and basal-cell carcinomas and of melanomas of the head, face and neck, but not of melanomas on other parts of the body. The high rate of lesions on the trunk and limbs in office workers may reflect their sunbathing or other recreational habits; but it contrasts clearly with other indoor work, where there is a generally low rate of all forms of skin cancer.


*Br. J. Cancer* (1981) **44,** 886

### THE RELATIONSHIP OF MALIGNANT MELANOMA, BASAL AND SQUAMOUS SKIN CANCERS TO INDOOR AND OUTDOOR WORK

V. BERAL AND N. ROBINSON
*From the Epidemiological Monitoring Unit, London School of Hygiene and Tropical Medicine, Keppel Street, London WC1E 7HT*

Received 27 April 1981 Accepted 27 August 1981

**Summary.—An analysis of occupational incidence data for malignant melanomas and squamous-and basal-cell carcinomas of the skin in England and Wales from 1970 to 1975 is reported. The occupational pattern for melanomas of the trunk and limbs differed markedly from the pattern for melanomas of the head, face and neck. Office work was associated with a large excess of melanomas of the trunk and limbs. In contrast, outdoor work was associated with an excess of melanomas of the head, face and neck; and was also associated with an excess of squamous-and basal-cell carcinomas of the skin. This suggests that prolonged occupational exposure to sunlight is an important cause of squamous-and basal-cell carcinomas and of melanomas of the head, face and neck, but not of melanomas on other parts of the body. The high rate of lesions on the trunk and limbs in office workers may reflect their sunbathing or other recreational habits: but it contrasts clearly with other indoor work, where there is a generally low rate of all forms of skin cancer.**

There is little doubt that prolonged exposure to sunlight, and UV light in particular, is a major cause of basal- and squamous-cell skin cancers (Urbach, 1978). Evidence for the role of sunlight in the aetiology of malignant melanoma is, however, less certain. Certain features of the epidemiology of melanoma, such as its increasing incidence as the equator is approached, and its predilection for fair-skinned red-haired people, implicate sunlight as a causal factor. On the other hand, there are inconsistencies in the evidence (Kripke, 1979; Anonymous, 1981).

Unlike other skin cancers, melanoma arises predominantly on the parts of the body not usually exposed to sunlight. Furthermore, available evidence suggests that melanomas arising on the normally covered parts of the body (the trunk and the limbs) are epidemiologically and histologically different from melanomas arising on the exposed parts of the body (the head, face and neck). Lesions of the trunk and limbs tend to occur at younger ages and their incidence is increasing more rapidly than melanomas of the head, face and neck (Ward, 1967; Magnus, 1977; Teppo *et al.*, 1978; Houghton *et al.*, 1980). In addition, a large proportion of melanomas of the head, face and neck are Hutchinson's melanotic freckles, which show evidence of solar degeneration in the surrounding skin, and are histologically different from melanomas arising elsewhere (McGovern *et al.*, 1980). Likewise basal- and squamous-cell carcinomas are frequently associated with solar degenerative changes such as solar keratoses (Gordon *et al.*, 1972). For these reasons it has been suggested that prolonged exposure to sunlight may be an important cause of squamous- and basal-cell carcinoma and of melanoma of the head, face and neck, but not of melanoma of the normally unexposed parts of the body (Ward, 1967).

Evidence about the relationship of occupational exposure to sunlight and melanoma is contradictory. Klepp & Magnus (1979) reported that working out of doors was associated with a small increase in melanoma in men but not women. In another case-control study Lancaster & Nelson (1957) found no such association. Analyses of vital statistical data consistently reveal high melanoma rates in professional and other occupational groups engaged in indoor office work (Lee & Strickland, 1980; Holman *et al.*, 1980). In contrast farmers and fishermen have melanoma mortality rates below the national average, suggesting that working out of doors is not necessarily associated with an increase in melanoma. Neither of these analyses systematically classified all occupations according to whether the work is carried out predominantly indoors or outdoors. Nor has the incidence of melanoma on different parts of the body been related to occupational exposure to sunlight.

In this paper we examine the relationship of place of work and risk of melanoma of the exposed and unexposed parts of the body separately. Because previous studies have indicated that office work, in particular, may be associated with an increased risk of melanoma, indoor workers were divided into office workers and other indoor workers. Incidence data for England and Wales from 1970 to 1975 were used. Basal- and squamous-cell skin cancers were included for comparison.

### METHODS

Data on melanoma (International Classification of Diseases (ICD) 172; 8th Revision, World Health Organisation, 1965) and other skin cancers (ICD 173) reported in England and Wales from 1970 to 1975 were obtained from the Office of Population Censuses and Surveys (OPCS). All persons with melanoma for whom an occupation was recorded were included. Because of the large numbers of basal-cell and squamous skin cancers, a 10% sample of the cancers where occupation was recorded was taken from each region. Details of the anatomical site of the melanomas were available from the 4th digit of the ICD code. Melanomas of "exposed" sites included lesions of the head, face and neck (ICD 172.0–172.4). Melanomas of the "unexposed" sites included other lesions (ICD 172.5–172.9). Occupation was coded according to the OPCS Classification of Occupations (1970). The occupation recorded for women was their own. Each individual was assigned on the basis of stated occupation, to 1 of 3 groups: outdoor workers, indoor office workers, and other indoor workers (mainly factory workers). Outdoor jobs included those where all or part of the work was performed out of doors, *i.e.* Occupational Units 001–010, 093–126, 129–135, 145–147, 151–153, 169, 171, 172. Indoor office jobs included Occupational Units 127, 128, 136–144, 148–150, 154–168, 170, 173–220. Other indoor jobs included Occupational Units 011–092. The classification system was designed such that, wherever possible, occupations were grouped into the OPCS Occupational Orders. This was because we wished to compare incidence with mortality data, and classifications no finer than Occupational Order are available for mortality from melanoma and other skin cancers. Mortality data from the Registrar General's Decennial Supplements on Occupation for 1961 and 1970–72 were used here. Outdoor occupations included Occupational Orders I, II and XV to XIX; indoor occupations included Occupational Orders XX to XXV; and other indoor occupations included Occupational Orders III to XIV. Social class was determined from the OPCS classification (1970).

Standardized cancer registration ratios (SRR) and standardized mortality ratios (SMR) were calculated by indirect standardization. The population in each occupational group in 1971 was used as a basis for the calculations (Registar General Decennial Supplement on Occupational Mortality 1970–72). The age-specific registration rates in all occupational groups combined was used as a standard for the calculation of SRR.

### RESULTS

Table I compares for men aged 15–64 years the distribution by place of work of the registered cases of melanoma and of other skin cancers. The occupational distribution is similar for squamous and basal-cell carcinomas and for melanomas of the head, face and neck, but the pattern for melanomas of other sites is quite different. There is a relative excess of squamous- and basal-cell carcinomas and melanomas of the head, face and neck in outdoor

TABLE I.—*Percentage distribution of registered cases of melanoma of exposed and unexposed sites and other skin cancers, by place of work. Men aged* 15–64 *years, England and Wales,* 1970–75

| | Outdoor work | Office work | Other indoor work | All occupations |
|---|---|---|---|---|
| Squamous and basal-cell carcinoma (numbers) | 37% (1194) | 38% (1221) | 25% (813) | 100% (3228) |
| Melanoma of the head, face and neck (numbers) | 36% (94) | 39% (104) | 25% (66) | 100% (264) |
| Melanoma of other sites (numbers) | 25% (285) | 50% (573) | 25% (281) | 100% (1139) |

TABLE II.—*Percentage distribution of registered cases of melanoma of exposed and unexposed sites and other skin cancers, by place of work. Women aged* 15–64 *years, England and Wales,* 1970–75

| | Outdoor work | Office work | Other indoor work | All occupations |
|---|---|---|---|---|
| Squamous and basal-cell carcinoma (numbers) | 8% (104) | 79% (1038) | 13% (166) | 100% (1308) |
| Melanoma of the face, head and neck (numbers) | 7% (8) | 82% (92) | 11% (12) | 100% (112) |
| Melanoma of other sites (numbers) | 6% (70) | 85% (938) | 9% (101) | 100% (1109) |

TABLE III.—*Standardized registration ratios for malignant melanoma of exposed and unexposed sites and other skin cancers, by place of work. Males aged* 15–64 *years, England and Wales,* 1970–75

| | Outdoor work | Office work | Other indoor work | All occupations |
|---|---|---|---|---|
| Squamous and basal cell carcinoma (numbers) | 110* (1194) | 97 (1221) | 92* (813) | 100 (3228) |
| Melanoma of the face, head and neck (numbers) | 109 (94) | 102 (104) | 87 (66) | 100 (264) |
| Melanoma of other sites (numbers) | 78* (285) | 131* (573) | 85* (281) | 100 (1139) |

* Differs significantly from 100 ($P < 0.05$)

workers. On the other hand there is a relative excess of melanomas of other sites in office workers. A generally similar pattern is evident for women, though it is less marked than for men (Table II).

Because the analyses in Tables I and II do not take the age structures of the populations in each occupational group into account, age-standardized registration ratios (SRR) were calculated). An SRR of 100 equals the national average. An SRR above 100 indicates a higher, and an SRR below 100 a lower disease rate than the national average. Table III shows the SRR for males aged 15–64, and it can be seen that the disease patterns in Table I persisted after standardization. Outdoor work was associated with a 10% excess of squamous- and basal-cell carcinoma and a 9% excess of melanomas of the head, face and neck, but a 22% deficit of melanomas of other sites. Office work was associated with a 31% excess of melanomas of other sites, and about average rates of squamous and basal cell carcinomas and of melanomas of the head, face and neck. In contrast to office workers, other indoor workers had a deficit of all tumour types. Since appropriate denominators for females classified by their own occupation are not available, it was not possible to calculate their SRR.

Because place of work may be confounded with social class, we repeated the

TABLE IV.—*Standardized registration ratios for malignant melanoma of exposed and unexposed sites and other skin cancers, by place of work. Males aged* 15–64 *years, England and Wales,* 1970–75. *Social Class III only*

| | Outdoor work | Office work | Other indoor work | All occupations |
|---|---|---|---|---|
| Squamous and basal cell carcinoma (numbers) | 112* (487) | 111* (391) | 85* (568) | 100 (1446) |
| Melanoma of the face, head and neck (numbers) | 105 (38) | 106 (31) | 81 (47) | 100 (116) |
| Melanoma of other sites (numbers) | 71* (111) | 143* (178) | 75* (189) | 100 (478) |

* Differs significantly from 100 ($P < 0.05$)

TABLE V.—*Standardized mortality ratio* (*SMR*) *for malignant melanoma and other skin cancers in* 1959–63 *and* 1970–72; *and standardized registration ratio* (*SRR*) *for the conditions in* 1970–75, *by place of work. Males aged* 15–64 *years, England and Wales*

| | Outdoor work | Office work | Other indoor work |
|---|---|---|---|
| Squamous and basal-cell carcinoma | | | |
| SMR 1959–63 (numbers) | 122* (134) | 89 (102) | 88 (69) |
| SMR 1970–72 (numbers) | 126* (74) | 68* (54) | 128* (68) |
| SRR 1970–75 (numbers) | 110* (1194) | 97 (1221) | 92* (813) |
| Melanoma | | | |
| SMR 1959–63 (numbers) | 90 (169) | 114* (235) | 102 (157) |
| SMR 1970–72 (numbers) | 92 (132) | 121* (244) | 87 (121) |
| SRR 1970–75 (numbers) | 94* (374) | 125* (677) | 85* (347) |

* Differs significantly from 100 ($P < 0.05$)

analyses in Table III, restricting the data to males aged 15–64 years in social class III (Table IV). The findings are similar to those in Table III, the main difference being that office work was also associated with a moderate increase in squamous and basal cell carcinoma (SRR = 111) and of melanomas of the head, face and neck (SRR = 106).

Our findings for cancer registration data were compared with those from mortality data (Table V). Melanomas of exposed and unexposed sites were combined, because occupational mortality data were not available by 4-digit ICD. For outdoor workers and indoor workers the SRR for 1970–75 and the SMR for 1959–63 and 1970–72 were very similar; there were a few inconsistencies for other indoor workers, but they generally showed a deficit of both tumour types.

### DISCUSSION AND CONCLUSION

Consistent with evidence from elsewhere on the carcinogenic effect of sunlight in squamous and basal-cell carcinomas, our analyses revealed that these tumours were most frequent in persons engaged in outdoor work. We found that melanoma of the exposed parts of the body (the head, neck and face) had a similar occupational distribution. In contrast, melanomas of the unexposed parts of the body were least common in persons engaged in outdoor occupations; but were especially common in those working in office jobs.

No similar comprehensive analysis of the relationship of skin cancer incidence to indoor and outdoor occupations has been undertaken before. Occupational incidence data are in many ways more representative than mortality data. In England and Wales, the male 5-year relative survival rate for melanoma is 48% and for other skin cancers is 99% (OPCS, 1980). Thus, a large number of incident cases may be missed by using mortality data alone. Furthermore, in Australia survival is better in the upper than the lower social classes (Shaw *et al.*, 1981). If this were so in England and

Wales, comparisons based on occupational mortality data would tend to underestimate the rates in the upper social classes. Another advantage of incidence data is that, with the large numbers involved, detailed analyses by site of lesion and place of work can be made. On the other hand there are disadvantages in their use, the main one being that occupation is not always recorded. Overall, about 75% of males aged 15–64 with registered melanoma had an occupation stated on their registration form; 71% of women had an occupation stated, but 54% of these were specified as "housewives" and not included in our analyses. Nor are all cancers registered. Under-registration is especially likely to occur with squamous and basal-cell cancers, as these lesions may be treated as an outpatient procedure and thus escape certain cancer registration schemes. But there is no reason to suspect that there are biases in reporting of tumours or the recording of occupation, depending on the site of the lesion. Thus, the under-reporting of occupation should not affect the comparisons made in this paper. Finally, incidence data are available over the 6-year period, 1970–75, but population estimates are available for 1971 only. Yet it is unlikely that the structure of the workforce changed in so short a time, such that the relative sizes of the populations working out of doors, in offices and in other indoor jobs were materially altered.

The findings of this study support other evidence that melanomas of the head, face and neck differ from melanomas of other parts of the body (Magnus 1977; Holman *et al.*, 1980; McGovern *et al.*, 1980). The similarity of the occupational patterns of melanomas of the head, face and neck with squamous- and basal-cell carcinomas strengthens arguments that prolonged exposure to sunlight is important in the aetiology of melanomas of the *exposed* parts of the body. On the other hand, the low incidence of melanomas of unexposed sites in outdoor workers indicates that occupational sunlight exposure does not increase the incidence of lesions on the normally covered parts of the body. Thus, the suggestion by Lee & Merrill (1970) that exposure of certain parts of the body to sunlight produces a circulating factor which increases the risk of melanoma on unexposed parts of the body is not supported by this evidence. The low rate of melanomas of the trunk and limbs in outdoor workers is perhaps not surprising, since outdoor workers in England and Wales do not generally expose those parts of their bodies to sunlight.

The most notable finding in our analysis is the high rate of melanomas on the unexposed parts of the body in office workers. This finding is particularly clear when data for a relatively homogeneous socio-economic group (social class III) are examined (Table IV). Although others have noted a high rate of all melanomas in office workers (Lee & Strickland, 1980), it has not been recognized before that this is because of an especially high rate of lesions on the unexposed parts of the body. Office workers contrast clearly with other indoor workers, who generally have below-average rates of melanomas, squamous- and basal-cell cancer. The reasons for the difference between office workers and other indoor workers is not clear. It is unlikely to be because of differential cancer registration, since similar patterns are generally noted in mortality data (Table V). Nor, as far as we can tell, is there a tendency to "upgrade" occupation by describing work as an office rather than another type of indoor job: when all cancers are taken together, those reported to be in office jobs had lower cancer registration rates and mortality rates than did those reported to be in other indoor jobs (Registrar General's Decennial Supplement on Occupation for 1961 and 1970–72). Office workers may be more likely than others to expose the normally covered parts of their bodies to sunlight, by sunbathing or in other leisure activities, or to take holidays in sunny countries (Magnus, 1973; Teppo *et al.*, 1978). Unfortunately there is no direct information available on the habits of office workers compared with others. However, even if they are more likely to be exposed to sunlight, it seems unlikely that prolonged exposure is an important aetiological factor in office workers.

The worldwide increase in melanoma incidence noted in the last 30 years is largely the result of an increase in lesions of the trunk and limbs (Magnus, 1977; Teppo *et al.*, 1978; Houghton *et al.*, 1980). There has been relatively little increase in melanomas of the head, face and neck. Since office workers experience high rates specifically of melanomas of the unexposed parts of the body, they should hold important clues to the recent increase in melanoma incidence.

We thank Ms Isobel Macdonald Davis from OPCS for providing the cancer registration listings, and Dr A. M. Adelstein and Mr Peter Smith for their comments on earlier drafts of the paper. The study was in part funded by the Medical Research Council.

### REFERENCES

ANONYMOUS (1981) The aetiology of melanoma. *Lancet* i, 253.

GORDON, D., SILVERSTONE, H. & SMITHURST, B. A. (1972) The epidemiology of skin cancer in Australia. In *Melanoma and Skin Cancer: Proc. Int. Cancer Conf.* Sydney: Blight Press, p. 23.

HOLMAN, C. D. J., MULRONEY, C. D. & ARMSTRONG, B. K. (1980) Epidemiology of pre-invasive and invasive malignant melanoma in Western Australia. *Int. J. Cancer*, **25**, 317.

HOUGHTON, A., FLANNERY, J. & VIOLA, M. V. (1980) Malignant melanoma in Connecticut and Denmark. *Int. J. Cancer*, **25**, 95.

KLEPP, O. & MAGNUS, K. (1979) Some environmental and bodily characteristics of melanoma patients: A case-control study. *Int. J. Cancer*, **23**, 482.

KRIPKE, M. L. (1979) Speculations on the role of ultraviolet radiation in the development of malignant melanoma. *J. Natl. Cancer Inst.*, **63**, 541.

LANCASTER, H. O. & NELSON, J. (1957) Sunlight as a cause of melanoma: A clinical survey. *Med. J. Aust.* i, 452.

LEE, J. A. H. & MERRILL, J. M. (1970) Sunlight and the aetiology of malignant melanoma: A synthesis. *Med. J. Aust.* ii, 846.

LEE, J. A. H. & STRICKLAND, D. (1980) Malignant melanoma: Social status and outdoor work. *Br. J. Cancer*, **41**, 757.

MCGOVERN, V. J., SHAW, H. M., MILTON, G. W. & FARAGO, G. A. (1980) Is malignant melanoma arising in a Hutchinson's melanotic freckle a separate disease entity? *Histopathology*, **4**, 235.

MAGNUS, K. (1973) Incidence of malignant melanoma of the skin in Norway, 1955–1970. *Cancer*, **32**, 1275.

MAGNUS, K. (1977) Incidence of malignant melanoma of the skin in five Nordic countries: Significance of solar radiation. *Int. J. Cancer*, **20**, 477.

OFFICE OF POPULATION CENSUSES AND SURVEYS (1970) *Classification of Occupations.* London: HMSO.

OFFICE OF POPULATION CENSUSES AND SURVEYS (1978) Occupational Mortality. *The Registrar General's Decennial supplement for England and Wales* 1970–72. *Series DS no.* 1. London: HMSO.

OFFICE OF POPULATION CENSUSES AND SURVEYS (1980) Cancer Statistics: Survival, 1971–73. *Series MB*1 *no.* 3. London: HMSO.

REGISTRAR GENERAL'S DECENNIAL SUPPLEMENT (1971) *Occupational Mortality Tables. England and Wales* 1961. London: HMSO.

SHAW, H. M., MCGOVERN, V. J., MILTON, G. W. & FARAGO, G. A. (1981) Cutaneous malignant melanoma: Occupation and prognosis. *Med. J. Aust.*, i, 60.

TEPPO, L., PAKKANEN, M. & HAKULINEN, T (1978) Sunlight as a risk factor for malignant melanoma of the skin. *Cancer*, **41**, 2018.

URBACH, F. (1978) Evidence and epidemiology of ultraviolet-induced cancers in man. *Nat. Cancer Inst. Monog.* **50**, 5.

WARD, W. H. (1967) Melanoma: Carcinoma of the skin and sunlight. *Aust. J. Dermatol.* **9**, 70.